# Analysis of the genetics of boar taint reveals both single SNPs and regional effects

**DOI:** 10.1186/1471-2164-15-424

**Published:** 2014-06-03

**Authors:** Suzanne J Rowe, Burak Karacaören, Dirk-Jan de Koning, Boris Lukic, Nicola Hastings-Clark, Ingela Velander, Chris S Haley, Alan L Archibald

**Affiliations:** The Roslin Institute and R(D)SVS, University of Edinburgh, Easter Bush, Midlothian, EH25 9RG Scotland, UK; Faculty of Agriculture, Department of Animal Science, Section of Biometry and Genetics, University of Akdeniz, Antalya, 07059 Turkey; The Department of Animal Breeding and Genetics, Swedish University of Agricultural Sciences, Uppsala, SE-750 07 Sweden; Faculty of Agriculture, University of J.J.Strossmayer in Osijek, Kralja Petra Svačića 1d, Osijek, 31000 Croatia; Pig Research Centre, Danish Agriculture & Food Council, Axeltorv 3, København, V 1609 Denmark; MRC Human Genetics Unit, MRC IGMM, University of Edinburgh, Crewe Road, Edinburgh, EH4 2XU Scotland, UK; AgResearch, Dept of Animal Genomics, Invermay Agricultural Centre, Private Bag 50034, Puddle Alley, Mosgiel, 9053 New Zealand

**Keywords:** Boar taint, Skatole, Androstenone, Regional heritability, Genome-wide association

## Abstract

**Background:**

Boar taint is an offensive urine or faecal-like odour, affecting the smell and taste of cooked pork from some mature non-castrated male pigs. Androstenone and skatole in fat are the molecules responsible. In most pig production systems, males, which are not required for breeding, are castrated shortly after birth to reduce the risk of boar taint. There is evidence for genetic variation in the predisposition to boar taint.

A genome-wide association study (GWAS) was performed to identify loci with effects on boar taint. Five hundred Danish Landrace boars with high levels of skatole in fat (>0.3 μg/g), were each matched with a litter mate with low levels of skatole and measured for androstenone. DNA from these 1,000 non-castrated boars was genotyped using the Illumina PorcineSNP60 Beadchip. After quality control, tests for SNPs associated with boar taint were performed on 938 phenotyped individuals and 44,648 SNPs. Empirical significance thresholds were set by permutation (100,000). For androstenone, a ‘regional heritability approach’ combining information from multiple SNPs was used to estimate the genetic variation attributable to individual autosomes.

**Results:**

A highly significant association was found between variation in skatole levels and SNPs within the *CYP2E1* gene on chromosome 14 (SSC14), which encodes an enzyme involved in degradation of skatole. Nominal significance was found for effects on skatole associated with 4 other SNPs including a region of SSC6 reported previously. Genome-wide significance was found for an association between SNPs on SSC5 and androstenone levels and nominal significance for associations with SNPs on SSC13 and SSC17. The regional analyses confirmed large effects on SSC5 for androstenone and suggest that SSC5 explains 23% of the genetic variation in androstenone. The autosomal heritability analyses also suggest that there is a large effect associated with androstenone on SSC2, not detected using GWAS.

**Conclusions:**

Significant SNP associations were found for skatole on SSC14 and for androstenone on SSC5 in Landrace pigs. The study agrees with evidence that the *CYP2E1* gene has effects on skatole breakdown in the liver. Autosomal heritability estimates can uncover clusters of smaller genetic effects that individually do not exceed the threshold for GWAS significance.

**Electronic supplementary material:**

The online version of this article (doi:10.1186/1471-2164-15-424) contains supplementary material, which is available to authorized users.

## Background

Boar taint is an offensive urine or faecal-like odour, affecting the smell and taste of some cooked pork. Androstenone and skatole, which are lipophilic compounds that accumulate in the fat of mature non-castrated male pigs, have been identified as the main causes of boar taint [1]. A range of thresholds, above which negative reactions from consumers are expected, have been reported for androstenone (>0.5-1.0 μg/g fat) and skatole (>0.2-0.25 μg/g fat) [2–6]. The scale of the problem was revealed in a large EU study of carcasses from over 40,000 non-castrated male pigs. Androstenone levels exceeded 1.0 μg/g fat and skatole levels exceeded 0.25 μg/g fat in 30% and 11% of these carcasses, respectively [3]. The cost of testing, losses in carcass value and potential future lost sales result in a substantial economic cost to the industry.

Androstenone or 5α-androst-16-en-one is a male steroid produced in the testes at sexual maturity. High concentrations of androstenone are present in the saliva of male pigs where it is converted to a pheromone and is an important olfactory trigger for sexual behaviour in sows [7]. Androstenone accumulates in adipose tissue producing taint when the fat is heated. The ability to detect this taint is itself under genetic control in humans and largely governed by the OR7D4 receptor. Approximately 70% of the human population are unable to detect the associated urine like odour [8, 9]. Skatole or 3-methyl-indole is produced from the breakdown of tryptophan by bacteria in the hindgut of the pig and subsequently absorbed into the blood stream where it is largely metabolised in the liver and excreted in urine. Skatole which is not degraded in the liver is deposited in peripheral tissues mainly accumulating in adipose tissue.

The most effective solution, to date, for controlling boar taint, is surgical castration shortly after birth. However, as castration removes natural anabolic androgens that promote lean growth, non-castrates are leaner with 10-30% greater efficiency in feed conversion and superior meat quality. Furthermore, concerns over animal welfare have led to legislative control [10]. Within Europe an industry-wide agreement is in place to cease castration for welfare reasons by 2018 (http://ec.europa.eu/food/animal/welfare/farm/initiatives_en.htm), forcing the industry to explore other methods to prevent tainted carcasses.

Selective breeding based on the identification and exploitation of genetic variation in androstenone and skatole levels could ultimately provide a more sustainable solution [11]. Recent studies have revealed Quantitative Trait Loci (QTL) with effects on skatole or androstenone, including QTL mapped to almost every chromosome [11–18]. The genetic architecture of predisposition to boar taint shows evidence for inter- and intra-breed variation with many of the reported effects appearing to be breed specific [11, 16–19]. In general, Duroc pigs tend to have high levels of androstenone, and the Landrace breeds high levels of skatole. The relationship between the two compounds is complex. Testicular steroids have been shown to inhibit the breakdown of skatole in the liver but the relationship between the compounds and the underlying mechanisms are not well understood [20].

Although highly successful at identifying new trait associated loci and pathways, human genome-wide association studies (GWAS) have failed to capture a large proportion of the genetic variation in complex traits [21, 22]. To address this so-called ‘missing heritability’ gap, methods have been developed involving the analysis of larger regions of the genome to account for variation unexplained by analysis of individual single nucleotide polymorphisms (SNPs) [23]. Estimating local heritability using larger regions captures additive variation in the genome which might elude the stringent significance thresholds necessary for testing each SNP individually. It has also been suggested that rare variants not in complete linkage disequilibrium (LD) with common SNP markers are captured by estimating the genetic variation from an entire “region” or set of SNPs [24].

The objective of this study was to identify genomic regions with effects on boar taint in Landrace pigs.

Results are reported from the two approaches used: (i) single SNP analysis using genome-wide association, and (ii) a regional approach dividing SNPs by chromosome and estimating genetic variation attributable to each autosome.

## Results

We acquired data for a population of approximately 6,000 commercial Danish Landrace boars. The animals were slaughtered at a mean age of 160 (±13) days. Measures for skatole were taken using an in-line procedure at three Danish abattoirs. Power to detect a QTL can be increased in a finite sample by selecting those individuals that differ most from the phenotypic mean i.e. the extremes of the phenotypic distribution. Here, we took extreme animals plus a within-litter ‘control’ in order to maximize power while controlling for family stratification. This strategy maximises the potential genetic information to be gained from the sample [25, 26]. Thus, 500 boars with high skatole (>0.3 μg/g fat) at slaughter, each matched with a low skatole litter mate (the lowest in the litter and in any event below 0.3 μg/g) were selected for genome-wide analysis. Phenotypic measurements for androstenone in adipose tissue were subsequently collected for these selected 1,000 boars.

The measures for both skatole and androstenone were positively skewed and were log transformed prior to analysis (Additional file 1: Figure S1). Descriptive statistics and heritabilities for both traits are given in Table 1. Pedigree information and skatole measures were available for 5,000 boars from the initial population that were not selected for genotyping and genome-wide analyses. Narrow sense heritabilities estimated from pedigree relationships h^2^_pedigree_ (LM 1) using all 6,000 records for skatole and 1,000 records for androstenone were moderate at 0.39 (s.e. 0.03) and 0.52 (s.e. 0.09) respectively and were similar to those previously reported [16, 27]. The genomic heritability estimate of 0.07 (s.e. 0.01) for skatole in the selected individuals was very low (Table 1). This result was expected and reflects the experimental design as the selected individuals comprised phenotypically divergent sibs for skatole thus maximising the within family variance. Narrow sense heritability is based on a ratio of the between and within family variance and is therefore reduced (and was similarly reduced in the pedigree based estimate using only the 1,000 genotyped individuals (not shown)). Comparing variance components estimated from the unselected and selected populations provides an indication of how effects estimated in the selected sample would scale to the population as a whole. Mean skatole measures for selected boars and their litter mates were 0.48 (sd. 0.25) and 0.15 (sd. 0.06) μg/g respectively. Although data were selected for skatole, androstenone measures also differed slightly (but not significantly) between the two groups with a mean of 1.25 (sd. 1.0) μg/g in the high skatole animals, and 0.85 (sd. 0.77) μg/g in their low skatole litter mates. The estimated genetic correlation between skatole and androstenone in the selected data was 0.27 (s.e. 0.20). Because the estimate of the additive genetic variance in skatole is biased downwards in the genotyped subset, the genetic correlation between skatole and androstenone is also likely to be underestimated.

**Table 1:** **Descriptive statistics for skatole and androstenone**

	Skatole	Androstenone
Mean (μg/g)	0.32	1.05
Sd (μg/g)	0.24	0.93
Range (μg/g)	0.02-2.49	0.06-9.23
Effect of slaughter weight (sig of effect)^Ϯ^	−0.0089 (3.43 E-05)	−0.023 (0.0007)
Effect of meat percentage (sig of effect)^Ϯ^	−0.048 (9.1 E-12)	−0.026 (0.06)
h^2^_pedigree_ (se)	0.39 (0.03)^ϮϮ^	0.52 (0.09)
h^2^_snp_ (se)	0.07 (0.01)	0.35 (0.08)

Data from 938 progeny of 128 sires and 441 dams.

^Ϯ^Covariate effects estimated in LMM using log trait.

h^2^_pedigree_ refers to narrow sense heritability estimated in a linear mixed model using GRM estimated from pedigree relationships.

^ϮϮ^Narrow sense heritability estimated for skatole using pedigree relationships from 6000 individuals.

h^2^_SNP_ refers to narrow sense heritability estimated in a linear mixed model using GRM estimated from SNP genotypes.

### Genome-wide association study (GWAS)

DNA isolated from muscle samples collected at slaughter were genotyped for 63,153 SNPs using the Illumina PorcineSNP60 beadchip [28]. Analysis was restricted to the autosomes. The genotype data were subjected to quality control (QC) through an iterative process performed using the GenABEL package in R 2.9.1 software [29, 30]. The QC criteria for SNPs were call rates > 0.95 and minor allele frequencies (MAF) > 0.01. The QC criteria for individuals were call rates > 0.95, heterozygosity > 0.45 (1% false discovery rate (FDR)) and identity-by-state (IBS) > 0.95. After QC, 44,648 autosomal SNPs and 938 individuals were included in the final analysis. SNP locations throughout the analysis are given according to the published draft pig genome sequence (Sscrofa10.2: ftp://ftp.ncbi.nlm.nih.gov/genbank/genomes/Eukaryotes/vertebrates_mammals/Sus_scrofa/Sscrofa10.2/) [31] and as available in Ensembl release 75 (http://www.ensembl.org/Sus_scrofa/Info/Index).

### Population stratification

**Figure 1:**
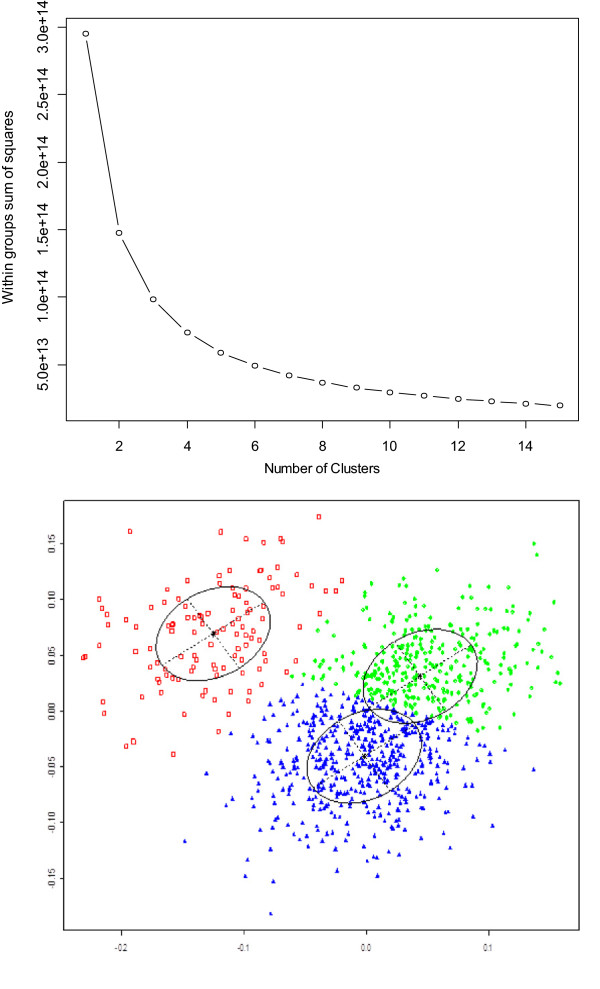
**Visualization of population structure.** Scree plot showing best fit shown by bend in curve is 3 clusters for the data (top). Plot of three clusters using co-ordinates from multi-dimensional scaling (bottom). Clusters are shown in green, red and blue. Individuals are assigned to clusters or groups based on degree of genetic relatedness.

Genome wide association is based on differences in allele frequencies associated with differences in the trait under study. Phenomena such as admixture, selection and population stratification can result in spurious patterns of allele frequencies unrelated to the trait. Population stratification can be assessed by clustering individuals based on measures of relatedness and examining clusters for evidence of systematic bias. Here, model based clustering was performed using the mclust function in R software 2.10.1 [30]. Mclust uses Bayesian information criterion (BIC) and an expectation maximisation algorithm (EM) to select the optimal model and number of clusters. The best fit for the data was 3 elipsoidal clusters (Figure 1). Multi-dimensional scaling (mds) was applied to a distance matrix obtained as a function of the weighted genomic relationship matrix. Multi-dimensional scaling returns a matrix with *k* columns whose rows give the coordinates of the points chosen to represent dissimilarities. *k* is a user defined parameter based on the expected number of clusters, here *k* = 3. The 3 columns from the mds matrix were fitted into the linear model as covariates in order to account for the population stratification indicated by the model based clustering.

The differences in study design between the two traits (i.e. skatole, androstenone) were reflected in the GWAS by very different estimates of lambda, which is an indicator of bias due to population structure. Lambda was close to 1 for all of the skatole analyses where high and low animals were matched sibs, but greater than 2 for the androstenone analyses. This result indicates that the sampling design for skatole was balanced and therefore, unaffected by potential biases arising from any population stratification. Although lambda indicates some bias for the androstenone analyses, this bias was largely accounted for with the inclusion of the co-ordinates from the multi-dimensional scaling (mds) matrix in the model (i.e. the inclusion of the mds matrix lowered the value of lambda from 2.0 to 1.3). Any remaining stratification was successfully corrected for by fitting the genomic relationship matrix. Full details are given in the materials and methods.

**Figure 2:**
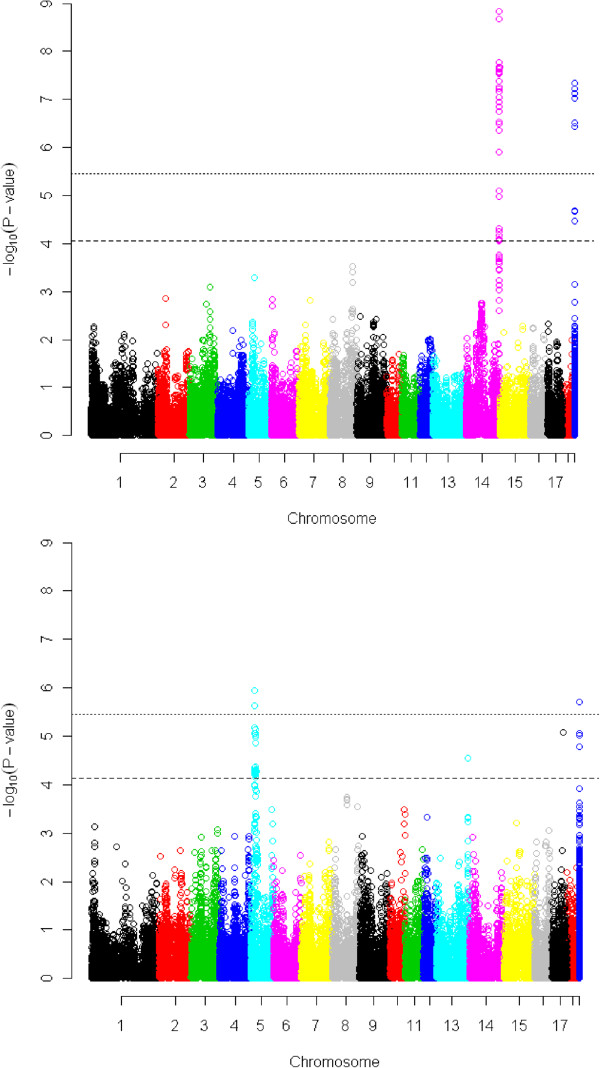
**Manhattan plots for genome-wide association analysis for associations with skatole (top) and androstenone (bottom).** Grammar method applied to eighteen autosomes plus unassigned SNPs (far right in dark blue). Genome-wide significance thresholds dashed line 5% FDR cut off. Dotted line is genome-wide significance threshold set by 100,000 permutations. Results are based on corrected P values using lambda statistic to account for systematic bias.

Single SNP associations were performed using a GRAMMAR [29] analysis (LM 3) in GenABEL software. The results are summarized in Figure 2. Test statistics exceeding genome-wide significance were found on SSC14 for skatole, and on SSC5 for androstenone. Further peaks on SSC13 and SSC17 exceed a genome-wide 5% FDR for effects on androstenone. Effects on skatole exceeding nominal significance but not genome-wide significance were also seen on SSC3, SSC5, SSC6 and SSC8 (Table 2).

**Table 2:** **Descriptive statistics for most significant SNP effects**

Chr	SNP	Pos (bp)§	P value	SNP effect	Proportion phenotypic variance	Sig-full^Ϯ^
	**Skatole**					
14	SIRI0000194	153,477,507	1.40E-09**	−0.26	0.05	1.66E-10
8	ASGA0039716	125,083,628	0.00029	0.04	0.001	0.0018
5	ASGA0025182	28,884,161	0.00052	0.12	0.02	0.00011
3	ALGA0020313	103,881,028	0.00082	0.17	0.01	0.0006
6	MARC0040638	4,515,061	0.00144	−0.13	0.01	0.00031
	**Androstenone**					
5	H3GA0016037	20,902,965	6.82E-07**	0.26	0.04	5.17E-07
5	ASGA0025097	24,354,867	3.51E-06*	0.28	0.03	2.03E-06
17	ASGA0095898	50,429,537	1.08E-05*	−0.52	0.02	0.0001
13	ALGA0073594	203,892,414	2.38E-05*	−0.17	0.02	3.63E-05
8	ASGA0093454	80,694,489	0.0002	−0.22	0.02	0.00024

*exceeds 5% genome-wide false discovery rate **exceeds genome-wide significance threshold estimated from 100,000 permutations ^Ϯ^significance when tested in linear mixed model using ASReml software. § SNP position in base pairs in the Sscrofa10.2 genome assembly.

### Skatole

The effect of the *SIRI0000194* SNP at the telomeric end of SSC14 on skatole levels was highly significant (*P* > 1.4E-09) exceeding the genome-wide threshold (Figure 2) and explaining ~5% of the phenotypic variance. This SNP lies within the *CYP2E1* gene, which encodes an enzyme involved in the breakdown of skatole [32–34]. The next ranking SNP after the SNPs in LD with *SIRI0000194* is the *ASGA0039716* SNP on chromosome 8. The *ASGA0039716* SNP lies within the gene *TET2* or *methylcytosine dioxygenase 2*. There is no obvious connection between the function of this gene or any other protein coding genes within 1 Mbp of *TET2* as currently annotated in the pig genome and skatole metabolism or storage. SNPs on chromosomes 3, 5 and 6 also reach nominal significance. When we fitted *SIRI0000194* as a fixed effect the ranking changed and *MARC0040638* was the top ranking SNP (*P* > 0.001).

### Androstenone

**Figure 3:**
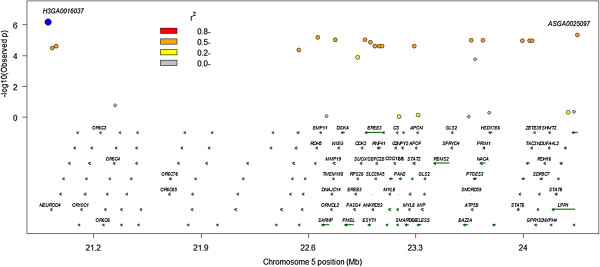
**LD decay from SNP** ***H3G000016037*** **plotted against significance of effect on androstenone, pairwise LD in the region and genes located within the region.** *Sscrofa* genome build 10.2.

A peak of genome-wide significant SNP effects on androstenone was seen on SSC5 (P > 6.8E-07) explaining 4% of the phenotypic variation (Table 2). Two SNPs *H3GA0016037* and *ASGA0025097* mapping 4 Mbp apart are highly significant. Figure 3 shows the LD structure and genes around the SSC5 peak SNP for androstenone. LD between the two SNPs is relatively high at r^2^ = 0.68 suggesting that both SNPs are tagging the same causal variant. There were also SNPs with large effects on chromosomes 8, 13 and 17 (Table 2). SSC13 and 17 exceeded the genome-wide false discovery rate. *ALGA0073594* on SSC13 does not map to any known gene. *ASGA0095898* on SSC17 lies within *PTPRT* or *protein tyrosine phosphatase, receptor type T* and *ASGA0093454* on SSC8 lies within the *FH2 domain containing 1* gene.

### Autosomal heritability

**Figure 4:**
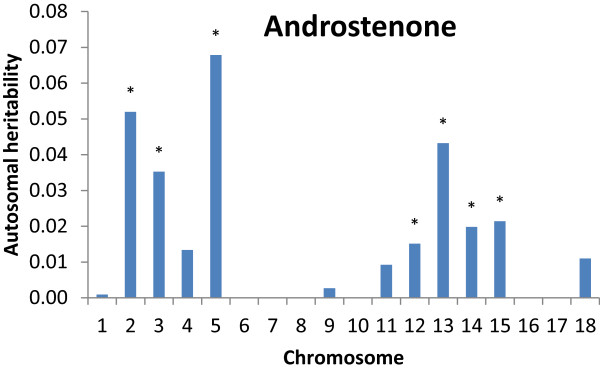
**Autosomal heritability or proportion of phenotypic variance explained for androstenone.** *estimate of heritability is larger than standard error. All 18 autosomes were fitted simultaneously in a mixed linear model.

The linear mixed model (2) can be extended to divide phenotypic variance into estimates of the genetic and environmental variance containing information from genotypes of a group of *N* SNPs spanning a region. This method has been implemented in the GCTA software package and it has been shown that the method can be used to estimate genetic variation for any region of the genome [35]. We divided the pig genome into the 18 autosomes and jointly estimated the contribution to heritability of androstenone (Figure 4, Additional file 2: Table S1) from each autosome (6). The total heritability summed over all autosomes was 0.29 for androstenone. As with the total heritability, the autosomal heritabilities for skatole will be specific to the genotyped subset and underestimated for the unselected population due to the study design. For this reason we have omitted the results on skatole from the main text, but these results can be found in Additional file 3.

Individual LRT (likelihood ratio tests) for each chromosome for androstenone are detailed in Table 3. These were derived by the LRT*poly* test comparing a linear mixed model fitting systematic or fixed effects and a GRM based on information from all SNPs with a model incorporating an additional variance component for the genetic variance attributable to all SNPs on a chromosome. This provides a test of whether inclusion of individual autosomes provides a better model of the variance than the overall relationship matrix (as might be the case if the individual chromosomes harbor a gene or genes of large effect on the trait). Estimates of the autosomal heritabilities for effects on androstenone for LRT*poly* are summarised in Table 3.

**Table 3:** **Estimates of autosomal heritability for androstenone**

Chr	h^2^_autosome_	se	***p***-val	h^2^_polygenic_	se
1	0	0.04	1	0.38	0.06
2	0.02	0.02	0.16	0.33	0.06
3	0.02	0.02	0.16	0.34	0.06
4	0	0.02	1	0.36	0.06
5	0.06	0.03	0.00051	0.29	0.06
6	0	0.03	1	0.37	0.06
7	0	0.02	1	0.36	0.06
8	0	0.02	1	0.36	0.06
9	0	0.02	1	0.37	0.06
10	0	0.02	1	0.36	0.06
11	0	0.02	1	0.36	0.06
12	0	0.02	0.38	0.35	0.06
13	0.02	0.02	0.21	0.33	0.06
14	0	0.02	1	0.36	0.06
15	0	0.02	0.36	0.35	0.06
16	0	0.02	1	0.36	0.06
17	0	0.02	1	0.36	0.06
18	0	0.01	1	0.36	0.06

Testing strategy was to compare fitting a random polygenic effect (based on a GRM estimated using all genotyped SNPs across the genome) plus a random effect for variance attributed to SNPs from a single autosome with a reduced model fitting only the random polygenic effect. *P*-val is the corresponding p value based on the distribution of the LRT being between χ^2^_1_ and a point mass of zero. h^2^ autosome is an estimate of the heritability of the autosome, h^2^ polygenic is an estimate of the heritability from the entire genome.

For androstenone, the only autosome with a significant LRT*poly* test for genetic variance was chromosome 5 explaining 6% of the phenotypic variation, reflecting the GWAS results. Under the LRT*poly* method, autosomes 2, 3 and 13 each explain 2% of the phenotypic variation, however, the estimates are not significant. When all autosomes are fitted simultaneously (Figure 4) SSC2, SSC3 and SSC13 explain 5%, 3% and 4% of the genetic variation. The sum of autosomal estimates of genetic variation from LRT*poly* is 0.12 (Table 3). The genetic variation explained by fitting all autosomes simultaneously was 0.29 (Additional file 2: Table S1), indicating that LRT*poly* is conservative as might be expected as part of the individual autosomal heritabilities are absorbed by the overall genomic polygenic effect.

**Figure 5:**
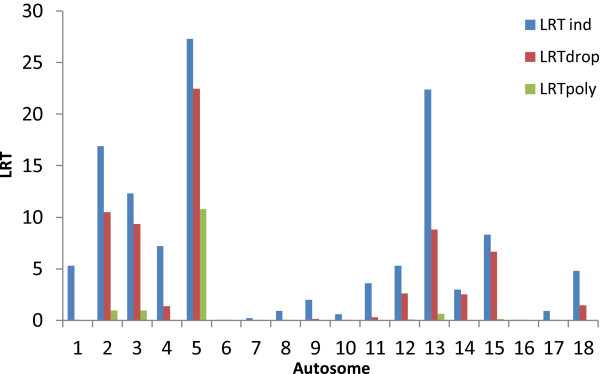
**Likelihood ratio test (LRT) for significance of autosomal heritability or proportion of phenotypic variance explained for androstenone using three different linear mixed models.** LRTind is comparing a model fitting an individual autosome with a null model, LRTdrop is where all autosomes are fitted and compared with a model which drops each autosome in turn, LRTpoly is comparing a model fitting an individual autosome plus a polygenic effect with a model containing only a polygenic effect.

An alternative testing strategy is to fit all autosomes in a full model and then drop them one at a time for a reduced model (LRT*drop*). A comparison of significance of autosomal heritability of androstenone using three testing strategies is given in Figure 5. Dropping a chromosome from the model including all the autosomes provides a test for whether genetic variance is associated with that particular chromosome whilst accounting for background polygenic effects on other chromosomes. This contrasts with the model containing only a single chromosome (LRT*ind* in Figure 5) where the LRT and variance explained may be inflated by genetic variance from the rest of the genome that is not explicitly included in the model. For androstenone the results for LRT*drop* suggest that chromosomes 2, 3, 5 and 13 explain a significant proportion of the variance.

## Discussion

**Figure 6:**
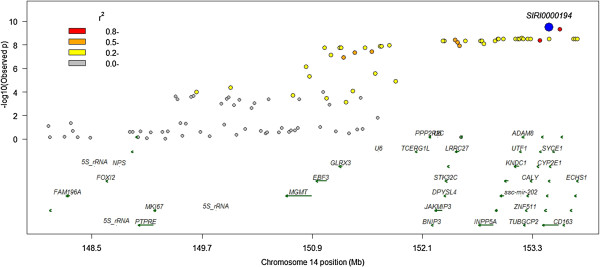
**LD decay from SNP** ***SIRI0000194*** **plotted against significance of effect on Skatole, pairwise LD in the region and genes located within the region.** *Sscrofa* genome build 10.2.

A genome-wide association study (GWAS) was carried out to identify SNPs associated with effects on androstenone and skatole in intact male pigs. The effect of the *SIRI0000194* SNP on skatole estimated by fitting the genotypes as a covariate in the linear mixed model (3) was 5% of the phenotypic variance of the selected population (Table 2). The expectation in the general population assuming a heritability of 0.4 is that it would explain ~12.5% of the genetic variation. The *SIRI0000194* SNP, which was reported previously as AJ697882_2412 [32], is located within the promoter of the *CYP2E1* gene. In a small separate sample of 83 Danish pigs significantly more AJ697882_2412 (*SIRI0000194*) *CC* homozygotes were observed in the ‘high’ skatole group [32]. More recently associations between skatole levels in two Duroc populations and the AJ697882_2412 (*SIRI0000194*) SNP have been reported [33]. Again the *CC* homozygotes exhibited the highest skatole levels. Although *SIRI0000194* lies within a block of high LD (Figure 6) spanning several other genes there is evidence to support *CYP2E1* as a candidate for the gene responsible for the observed associations with skatole levels. This gene has been previously identified as a candidate and is involved in the degradation of skatole in the liver where it is solely and abundantly expressed [36] (see also (http://biogps.org).

The GWAS for skatole was repeated, fitting the SNP *SIRI000094* into the linear mixed model as a fixed effect. This model resulted in a change of ranking among the SNPs. The effect of greatest significance (P > 0.001) was associated with SNP marker *MARC0040638* located on chromosome 6 within the *estradiol 17-beta-dehydrogenase 2 (HSD17B2)* gene. The *HSD17B2* gene and *MARC0040638* SNP were located at SSC6:4,514,200-4,578,665 in an earlier genome assembly (Sscrofa9) but are located on unplaced scaffolds on the present assembly (Sscrofa10.2). The assignment of *MARC0040638* SNP to SSC6 is confirmed from radiation hybrid mapping data (Additional file 2: Table S1 in [37]). Both the *MARC0040638* SNP and *HSD17B2* gene are present in the sequence of the CH242-77H3 BAC clone (Genbank accession: CU929847). Incomplete sequence data from this BAC clone contribute to the current pig genome assembly (Sscrofa10.2) on SSC6 6.876-6.939 Mbp. This SNP did not exceed the FDR or genome-wide threshold, however a region on chromosome 6 spanning this gene was previously found to be significant for skatole in Landrace pigs [18]. Ramos et al., [18] found significant associations between skatole levels in Duroc pigs and SNPs mapping to a 6 Mbp region on SSC6 corresponding to 1.829-8.498 Mbp in Sscrofa10.2 coordinates and thus including the *MARC0040638* SNP and *HSD17B2* gene. In an earlier study, we mapped QTL for skatole, as detected by a (human) sensory panel, by linkage analysis with a low density microsatellite marker map with the closest marker *SW1353* mapping to SSC6: 9.872 Mbp (Sscrofa10.2 coordinates) [13]. Human *estradiol 17-beta-dehydrogenase 2 (HSD17B2)* is involved in the synthesis of the 17 beta-hydroxysteroids: delta 5-androstene-3 beta, 17 beta-diol, testosterone, 17 beta-estradiol and dihydrotestosterone [38]. The *HSD17B2* gene is thus important for steroid hormone synthesis and is abundantly expressed in pig liver, ureter and stomach (fundus), [36] (see also (http://biogps.org). Another *17-beta hydroxysteroid dehyrdorgenase gene (HSD17B7)* has been examined as a candidate gene for an androstenone QTL on SSC4 [39].

A significant effect on androstenone was found associated with the *H3GA0016037* SNP on chromosome 5 explaining ~4% of the phenotypic variance. *H3GA0016037* lies between the gene encoding transcription factor *NEUROD4 neurogenic differentiation 4* and the *TESPA1 thymocyte expressed positive selection association 1* locus. In humans *TESPA1* is involved in the selection of thymocytes and T-cell development. It has been hypothesised that the production of glucocorticoid steroids may in some way regulate thymocyte selection [40]. The second most significant GWAS result was for *ASGA0025097* which is located ~4 Mbp distal to the *H3GA0016037* SNP on chromosome 5. The genes of interest located within this 4 Mbp region include the *retinol dehydrogenase 5 (RDH5)* and *retinol dehydrogenase 16 (RDH16)* genes. The *RDH* gene encodes an enzyme which recognizes 5α-androstan-3α,17β-diol and androsterone as substrates and is expressed in liver, testes and other tissues in humans [41]. *RDH16* is abundantly expressed in pig liver, testes and placenta [36] (see also http://biogps.org). Another *17-beta hydroxysteroid dehyrdrogenase* gene (*HSD17B6*) is located about 0.5 Mbp upstream of the *ASGA0025097* SNP. The 4 Mbp region between the two top SNPs is gene-rich and exhibits high levels of LD in the Danish Landrace population studied (Figure 3). Ironically, many of the genes in this region encode olfactory receptors. The minor allele frequency for both SNPs (*ASGA0025097, H3GA0016037*) was 0.14 and the r^2^ between them was 0.68. Fitting either SNP results in the loss of the effect indicating that both SNPs are tagging the same causal variant. This region has been found to be significant for androstenone measured in the fat of Duroc pigs, and for estradiol in Landrace pigs [16], however this region has not previously been found to be significant for effects on androstenone levels in Landrace.

Results from the regional heritability study reflected the GWAS analysis with the greatest heritability for androstenone on chromosome 5. This indicates that the regional approach successfully identifies autosomes with genetic variation attributable to the trait and that genetic variance is not correlated to the length of autosomes as seen by Yang et al. [24]. Here, the correlation of variance explained, with length of autosome, was 0.02 (P > 0.93) for androstenone. There was evidence of information beyond the GWAS results from the regional approach. The method did point to an association of SSC2 and SSC3 with androstenone not seen in the GWAS. Highly significant effects for multiple QTL on these chromosomes associated with androstenone have been previously reported [11–13, 16]. We cannot ascertain whether the SNP effect on SSC17 associated with androstenone is undetected by the regional approach or a spurious artifact of the GWAS. One approach might be using sequence information for imputation to increase the number of SNP genotypes and subsequently to divide the genome into many smaller regions providing greater resolution. Combined results of multiple SNP genotypes are less likely to yield spurious results from anomalies such as population stratification and differing minor allele frequencies at individual SNPs. The autosomes explaining the most variation have a greater likelihood for housing putative candidate genes and pathways. A further use for the estimated SNP or region effects in this population could be genomic prediction in unphenotyped individuals. This potential application is of particular relevance in traits that can only be measured post slaughter such as boar taint where phenotypes are of high economic impact and could result in rejection of the entire carcass.

## Conclusions

Significant associations were found for skatole on SSC14 and for androstenone on SSC5 in Landrace pigs. The study agrees with a body of evidence that the *CYP2E1* gene has effects on skatole breakdown in the liver. Autosomal heritability estimates agree with the GWAS and provide an opportunity to identify regions for further study. Differences between the GWAS and the autosomal heritability suggest that for androstenone there is variation explained by SSC2 and SSC3 that is not detected by the GWAS and that the SNP on chromosome 17 does not appear to contribute variance at the level of the autosome.

## Methods

### Animals

All the animals involved in this study were raised under conventional pig production conditions and were not subjected to any experimental procedures. All the samples for the study were collected post-mortem in a commercial abattoir.

### Taint measures

Tissue fat samples were assayed for skatole levels using a calorimetric method in-house at the abattoir [42]. A second tissue sample taken about an hour after slaughter was subsequently assayed for androstenone by the Norwegian School of Veterinary Science using a modified time-resolved fluoroimmunoassay [43].

### Heritabilities

A fixed effect of herd; and significant covariates meat percentage, slaughter weight and age at slaughter, were estimated using a linear mixed model in software package ASReml2 [44] (1). Fixed effects and covariates for skatole were estimated using the entire population of 6,000 animals in order to achieve the greatest possible accuracy. Heritabilities were estimated using pedigree relationships in the entire population of 6,000 individuals for Skatole and the 1,000 individuals phenotyped for androstenone.1

Where **Y** is an *n* × 1, vector of log phenotype, *n* is the number of individuals, **X** is an incidence matrix relating solutions for fixed effects of herd and covariates of age, mds co-ordinates contained in **β** to individuals, **u** is an *n* × 1 vector of genetic effects, **Z** is an *n × n* incidence matrix relating individuals to genetic effects, and **e** is an *n* × 1 vector of individual residual effects. , and **e** is distributed as **e** ~ N(0, **I***σ*^2^_e_). **A** is the *n × n* genetic relationship matrix estimated from pedigree relationships.

### Genomic relationship matrices

SNP genotypes were used to estimate shared coancestry or identity by state between individuals with rare SNPs weighted more heavily. The *n × n* genomic relationship matrix (GRM) of relatedness at a population level between *n* individuals gives the covariance structure for the phenotype based on the premise that the more related two individuals are, or the greater the amount of the genome they share in common, the greater the expectation of phenotypic similarity. The proportion of alleles two individuals share in common are summed across all markers weighted by allele frequencies in the population in order to obtain an accurate estimate of how related two individuals are either across the entire genome or at a given region. Genomic relationship matrices were estimated using GenABEL [29] and GCTA [35] software.

Using the marker information for the 1,000 individuals, heritabilities were estimated by fitting the SNP based genomic relationship matrix from GenABEL in a linear mixed model to estimate polygenic effects from marker information (2). A genotypic correlation was estimated by a bivariate analysis of the two traits fitting the genomic relationship matrix using ASReml 2 software [44].2

Where **g** is an *N* × 1 vector of SNP effects, *N* is the number of SNPs, **W** is an *n × N* incidence matrix relating SNP genotypes to **g. G** is the *n × n* genomic relationship matrix estimated from SNP genotypes and .

### Association analysis

Single SNP association tests were performed using a GRAMMAR [29] analysis (3) in GenABEL software. GRAMMAR uses a score test to identify associations between SNP genotypes and trait residuals after fixed and background genetic or polygenic effects are accounted for in the linear mixed model (2). Polygenic effects were estimated using a grm estimated from the average relationship between individuals at all SNP markers (weighted by allele frequency) across the genome.34

Where T is test statistic for *N* SNPs from (3).

Where **y** is a vector of trait residuals from (2), **SNP** is a vector of SNP genotypes and **e** is a vector of random residuals.

A correction factor or lambda [29, 45] was estimated from the distribution of test statistics to further account for systematic bias (4). A factor greater than 1 is indicative of systematic inflation of test results when compared to a distribution of the expectation under the null hypothesis. A factor less than one often results from over correction in a grammar analysis. The grammar function in GenABEL adjusts for this deflation factor. Permutation analysis (100,000) was used to determine a rigorous threshold for genome-wide significance accounting for multiple testing and for any unaccounted for systematic bias. A less rigorous FDR cut off of >0.05 was applied to report SNPs of interest to aid the comparison of results from past and future study populations.

As grammar analyses tend to underestimate true SNP effects [29], genome-wide significant SNPs identified with the grammar analysis were fitted individually as covariates in the linear mixed model using ASReml 2 software to estimate SNP effects and to verify significance (5). The additive genetic variance was estimated as 2*p*(1-*p*)*α*^2^ where *p* is the allele frequency for the most common SNP allele and α is the estimated effect. A further check was that this estimate was consistent with the difference in phenotypic variance when fitting, and not fitting, SNP genotype as a covariate in the LMM.5

Where y = log trait. Herd is fitted as a fixed effect. SNP genotype, slaughter weight, age, meat percentage and co-ordinates from the multi-dimensional scaling (mds) are fitted as covariates, *a* is a random polygenic effect estimated using a SNP-based relationship matrix and e is the random residual.

### Estimation of regional genetic contribution or ‘autosomal heritability’

The linear mixed model (2) can be extended to divide phenotypic variance into estimates of the genetic and environmental variance containing information from genotypes of a group of *N* SNPs spanning a region. This method has been implemented in the GCTA software package and it has been shown that the method can be used to estimate genetic variation for any region of the genome [35]. We divided the pig genome into the 18 autosomes and estimated the contribution to heritability from each autosome (6). For these analyses only SNPs that mapped to Sscrofa 10.2 were used, any SNPs without a position on the current assembly were omitted as they could not be assigned to an autosome. Omitting these SNPs (~13% of all SNPs) from the GRM made very little difference to the estimate of total genetic variance. The heritability estimate dropped by 0.0065. This indicates that this subset of annotated SNPs was sufficiently large enough to accurately estimate relationships between individuals and to capture the genetic variance.6

To avoid confounding of genetic variation of the trait and potential variation due to population stratification, eigenvectors were estimated from the genetic relationship matrix and the first 4 principal components fitted as covariates in the linear mixed model. This is slightly conservative and based on the results of the model based clustering described earlier which showed that the data forms 3 distinct clusters. The fixed effects and covariates of herd, age, meat percentage and slaughter weight were fitted into a linear mixed model together with eighteen variance components - one for each of the eighteen autosomes requiring 18 separate genetic relationship matrices to model the covariance structure and to partition the genetic variance into estimates of autosomal heritability.

To test the significance of individual autosomes a likelihood ratio test (LRT) comparing a model fitting the individual autosome plus a variance component for all SNPs in the grm (i.e. the equivalent of a genomic polygenic effect) was compared to a model fitting only the polygenic effect (LRT*poly*). All SNPs were used in the polygenic effect to ensure that the models were truly nested. This conservative approach ensures that the variance explained by an autosome is not inflated by background polygenic effects.

Two further approaches were used. Firstly, comparing a model fitting a variance component estimated from the SNPs on a single autosome with a null model (LRT*ind*). Secondly, a model fitting all 18 variance components compared with a model dropping each of the autosomes in turn (LRT*drop*).

GCTA solves the linear mixed model (LMM) and obtains estimates of genetic and residual variances by restricted maximum likelihood (REML) using the average information (AI) algorithm.

A test statistic was obtained using a standard LRT statistic calculated as twice the difference between the log likelihoods of the full model and the null or reduced model that did not fit a genetic component. The LRT was tested against a chi square distribution. The LRT for one extra variance component is distributed as a mixture of point 0 and 1degrees of freedom (df) [46]. To account for this a *P*-value for a test assuming 1df was divided in two.

## Electronic supplementary material

Additional file 1: Figure S1: Distribution of skatole and androstenone measures and log transformed measures of skatole and androstenone. (PDF 70 KB)

Additional file 2: Table S1.: Effect of testing structure on estimates and significance of autosomal heritability. (PDF 52 KB)

Additional file 3:: **Autosomal heritability analysis for skatole.** (PDF 95 KB)
