# Supplementary material for: Analysis of the genetics of boar taint reveals both single SNPs and regional effects
Source: BMC Genomics. 2014 Jun 3;15(1):424. doi: 10.1186/1471-2164-15-424 (PMC4059876; doi:10.1186/1471-2164-15-424)
Supplement: Supplementary file 1 — Additional file 1: Figure S1: Distribution of skatole and androstenone measures and log transformed measures of skatole and androstenone. (PDF 70 KB) [file 12864_2013_6115_MOESM1_ESM.pdf]

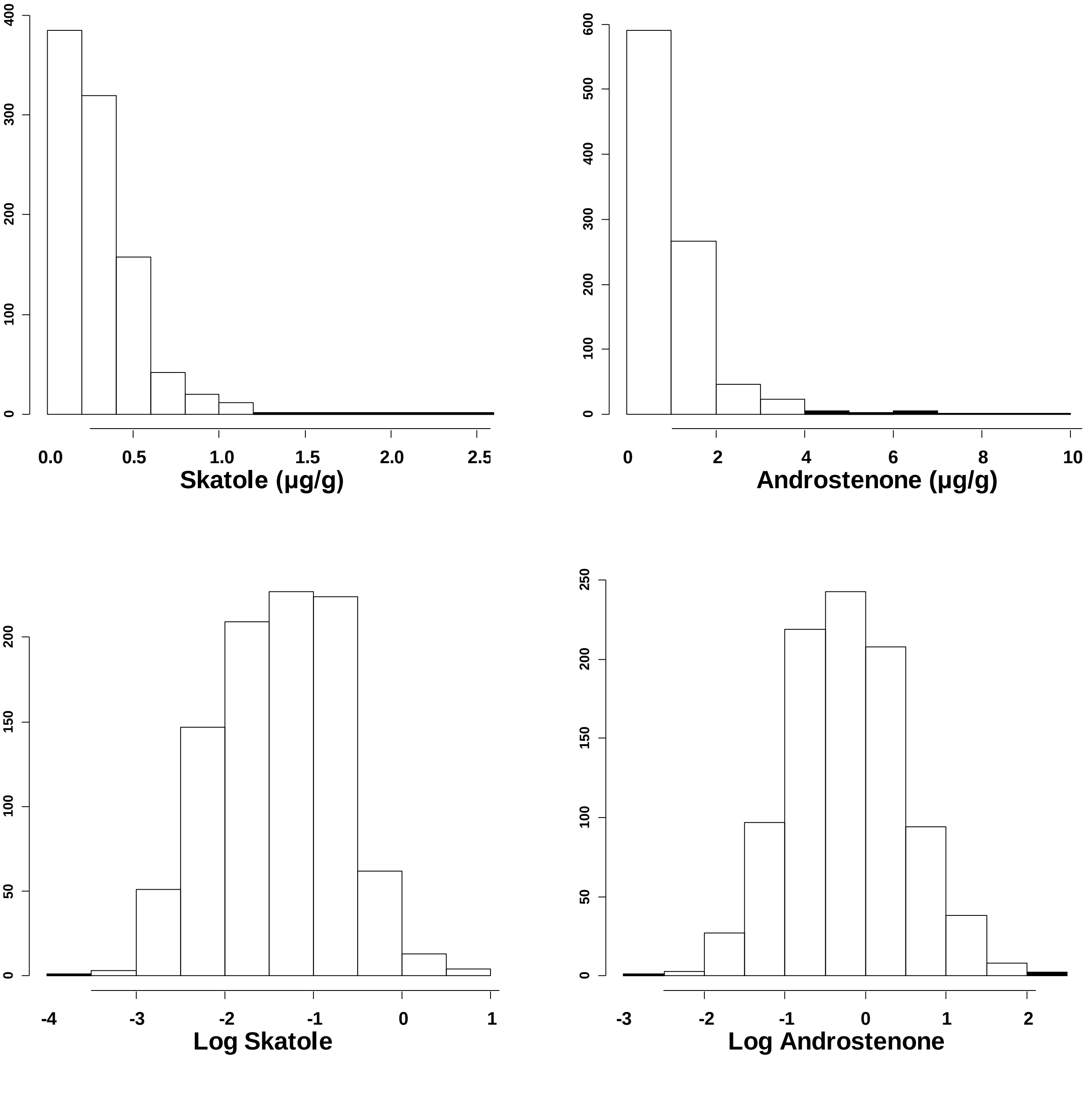

Supplementary Figure 1 Histograms of skatole (left) and androstenone (right), untransformed (top) and log transformed data (bottom).
