# Supplementary material for: Analysis of the genetics of boar taint reveals both single SNPs and regional effects
Source: BMC Genomics. 2014 Jun 3;15(1):424. doi: 10.1186/1471-2164-15-424 (PMC4059876; doi:10.1186/1471-2164-15-424)
Supplement: Supplementary file 2 — Additional file 2: Table S1.: Effect of testing structure on estimates and significance of autosomal heritability. (PDF 52 KB) [file 12864_2013_6115_MOESM2_ESM.pdf]

Supplementary Table 1:

Effect of testing structure on estimates and significance of autosomal heritability.

			Androstenone				
Chr	LRT	p-val	h^2_{ind}	se	h^2_{all}	se	%herit
1	5.3	0.0208	0.063	0.032	0.001	0.014	0.32
2	16.9	3.99E-05	0.064	0.027	0.052	0.024	17.8
3	12.3	0.0004	0.051	0.025	0.035	0.02	12.08
4	7.2	0.0072	0.042	0.023	0.013	0.015	4.58
5	27.3	1.78E-07	0.087	0.03	0.068	0.026	23.23
6	0.02	0.8993	0.002	0.012	0	0.011	0
7	0.2	0.639	0.008	0.016	0	0.013	0
8	0.9	0.3297	0.017	0.018	0	0.013	0
9	2	0.1613	0.029	0.022	0.003	0.015	0.93
10	0.6	0.4332	0.01	0.015	0	0.011	0
11	3.6	0.057	0.034	0.022	0.009	0.016	3.17
12	5.3	0.0212	0.029	0.019	0.015	0.014	5.18
13	22.4	2.17E-06	0.065	0.026	0.043	0.023	14.81
14	3	0.0845	0.025	0.02	0.02	0.018	6.8
15	8.3	0.004	0.033	0.02	0.021	0.015	7.33
16	0.02	0.87	0.002	0.011	0	0.011	0
17	0.9	0.3509	0.013	0.015	0	0.01	0
18	4.8	0.0279	0.038	0.023	0.011	0.014	3.77
Total			0.61		0.292		

LRT is the likelihood ratio test of a model fitting a random effect for variance attributed to SNPs from an autosome compared with a null model. Pval is the corresponding p value based on the distribution of the LRT being between X^2_1 and a point mass of zero. h^2_{ind} is an estimate of the heritabilty of an autosome where the autosome has been fitted individually in a linear mixed model. h^2_{all} is an estimate of heritability for an autosome from a model where all 18 autosomes have been fitted simultaneously in a linear mixed model.
