# Supplementary material for: Analysis of the genetics of boar taint reveals both single SNPs and regional effects
Source: BMC Genomics. 2014 Jun 3;15(1):424. doi: 10.1186/1471-2164-15-424 (PMC4059876; doi:10.1186/1471-2164-15-424)
Supplement: Supplementary file 3 — Additional file 3:: Autosomal heritability analysis for skatole. (PDF 95 KB) [file 12864_2013_6115_MOESM3_ESM.pdf]

Supplementary file 1

Autosomal heritability analyses for skatole

Use of a linear mixed model to estimate heritability is based on an unbiased sample of the population. It is assumed that family members are more likely to share alleles identical-by-descent (IBD) and therefore for an inherited trait genetic variance is expected to be much greater between families than within. A discordant sib pair design maximizes within family variance. This is a powerful way to differentiate between siblings that inherit alleles at an individual locus as the phenotype can be regressed on the absolute proportion of alleles shared between full sibs, i.e. 0, 0.5 or 1. This design, however, provides an estimate of the whole genome or polygenic variance component that is biased downwards. This bias arises because the GRM used to model covariances amongst relatives is based on summation of the proportion of alleles shared at each locus. This bias is shown clearly where for skatole, heritability estimated in the sample population of 6,000 individuals is 0.35, but heritability estimated from 500 discordant sib pairs selected from the same population is 0.07. For the autosomal heritability analyses described here, bias remains a factor although we expect estimates to be proportional to the true variance. Others have tried to scale this bias (Chamberlain AJ, Duc LT, Bowman PJ, Goddard ME: Unbiased estimates of variances due to QTL detected by selective genotyping. Proc Adv Anim Breed Gen 2003, 15; http://livestocklibrary.com.au/handle/1234/5604) but it is difficult to estimate. The design does facilitate, however, the detection of regions in high LD that are correlated with the phenotype when the GRM is estimated using only SNPs from this region. This may be reflected at the level of the autosome. We have therefore omitted the estimates from the main manuscript but present them here for completeness.

Method

A regional heritability approach combining information from multiple SNPs was used to estimate the genetic variation attributable to individual autosomes. Autosomes were fitted simultaneously in a linear mixed model to partition genetic contribution from each chromosome. The full analyses are described in the materials and methods section of the manuscript.

Results

We divided the pig genome into the 18 autosomes and estimated the contribution to heritability of skatole from each autosome (Figure S2).

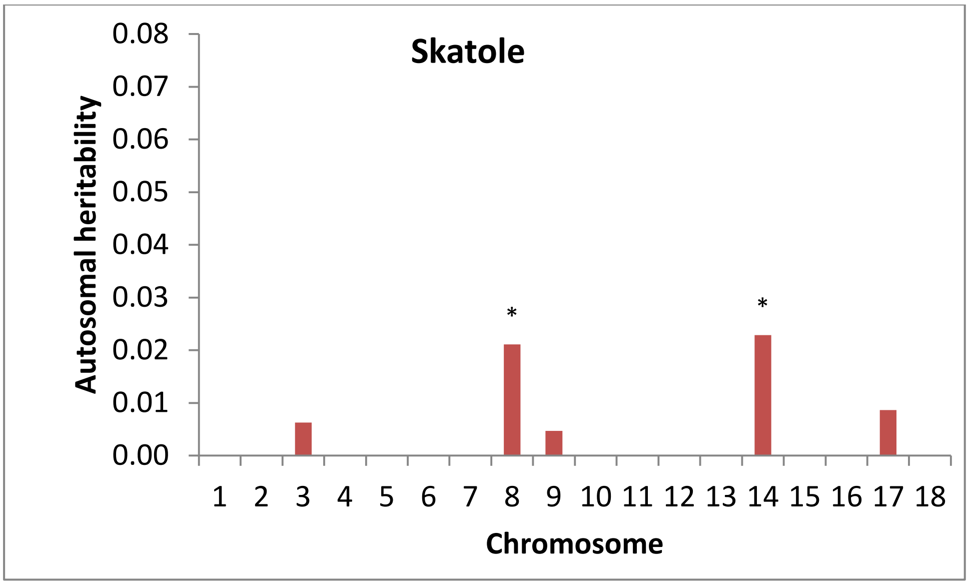

Figure S2: Autosomal heritability or proportion of phenotypic variance explained for skatole. *estimate of heritability is larger than standard error. All 18 autosomes were fitted simultaneously in a mixed linear model.

The total heritability of Skatole summed over all autosomes was 0.06. Individual test statistics for each autosome are detailed in Table S2. For skatole, significant estimates of genetic variance for skatole were confined to SSC8 and SSC14. These autosomal heritability results reflect those from the GWAS analyses with 4% of the phenotypic variation in skatole explained by the SNPs on chromosome 14. Furthermore, although there is insufficient evidence in the GWAS to conclude that SNP *ASGA0039716* on chromosome 8 is associated with skatole, the autosomal heritability estimates indicate that the variance explained by chromosome 8 is almost as great as that explained by chromosome 14. We believe that these estimates, although biased, are proportional to the true variance in the population and therefore indicate that autosomes 8 and 14 contain regions controlling the genetic variance of skatole.

Table S2: Estimates of autosomal heritability for Skatole

Chr	$h^2_{autosome}$	se	*p*-val	$h^2_{polygenic}$	Se
1	0	0.02	1	0.04	0.04
2	0	0.02	1	0.04	0.04
3	0	0.01	0.41	0.04	0.04
4	0	0.01	1	0.04	0.04
5	0	0.01	1	0.04	0.04
6	0	0.02	1	0.04	0.04
7	0	0.01	1	0.04	0.04
8	0.03	0.02	0.04	0.02	0.04
9	0	0.01	1	0.04	0.04
10	0	0.02	1	0.04	0.04
11	0	0.02	1	0.03	0.04
12	0	0.01	0.37	0.04	0.04
13	0	0.02	1	0.04	0.04
14	0.04	0.02	0.000052	0	0.04
15	0	0.01	1	0.04	0.04
16	0	0.01	1	0.04	0.04
17	0	0.01	1	0.04	0.04
18	0	0.01	1	0.05	0.04

The testing strategy was to compare fitting a random polygenic effect (based on a GRM estimated using all genotyped SNPs across the genome) plus a random effect for variance attributed to SNPs from a single autosome with a reduced model fitting only the random polygenic effect. *P*-val is the corresponding p value based on the distribution of the LRT being between χ^2_1 and a point mass of zero. h^2 autosome is an estimate of the heritability of the autosome, h^2 polygenic is an estimate of the heritability from the entire genome.

We compared autosomal heritability estimates from models in which the effects of individual autosomes were estimated separately with one in which they were all estimated jointly (Table S3).

Table S3: Effect of testing structure on estimates and significance of autosomal heritability.

				Skatole			
Chr	LRT	Pval	h^2 ind	se	h^2_{all}	se	%herit
1	0.1	0.7	0.005	0.014	0	0.014	0
2	0	1	0	0.016	0	0.016	0
3	0.3	0.58	0.006	0.013	0.006	0.014	9.82
4	0	1	0	0.01	0	0.011	0.14
5	0	1	0	0.013	0	0.014	0
6	0	1	0	0.013	0	0.016	0
7	0.01	0.9	0.001	0.012	0	0.013	0
8	3.9	0.05	0.036	0.023	0.021	0.012	33.13
9	0.03	0.84	0.002	0.012	0.005	0.016	7.36
10	0	1	0	0.014	0	0.017	0
11		1			0	0.015	0
12	0.29	0.59	0.005	0.011	0	0.01	0
13	0	1	0	0.012	0	0.014	0
14	17	3.76E-05	0.06	0.026	0.023	0.015	35.94
15	0	1	0	0.012	0	0.015	0
16	0	1	0	0.01	0	0.011	0
17	0	0.87	0.002	0.011	0.009	0.014	13.6
18	0	1	0	0.008	0	0.009	0
Total			0.118		0.064		

LRT is the likelihood ratio test of a model fitting a random effect for variance attributed to SNPs from an autosome compared with a null model. Pval is the corresponding p value based on the distribution of the LRT being between X^2_1 and a point mass of zero. h^2_{ind} is an estimate of the heritabilty of an autosome where the autosome has been fitted individually in a linear mixed model. h^2_{all} is an estimate of heritability for an autosome from a model where all 18 autosomes have been fitted simultaneously in a linear mixed model.
